# Sensitivity Evaluation of a Dual-Finger Metamaterial Biosensor for Non-Invasive Glycemia Tracking on Multiple Substrates

**DOI:** 10.3390/s25227034

**Published:** 2025-11-18

**Authors:** Esraa Mansour, Mohamed I. Ahmed, Ahmed Allam, Ramesh K. Pokharel, Adel B. Abdel-Rahman

**Affiliations:** 1 Electronics and Communications Department, Egypt-Japan University of Science and Technology, Alexandria 21934, Egypt; ahmed.allam@ejust.edu.eg (A.A.); adel.bedair@ejust.edu.eg (A.B.A.-R.); 2 Microstrip Department, Electronics Research Institute, Joseph Tito St, Huckstep, El Nozha, Cairo 12622, Egypt; 3 Faculty of Engineering and Technology, Egyptian Chinese University, Cairo 4541312, Egypt; 4 Graduate School of Information Science and Electrical Engineering, Kyushu University, Nishi Ku, Fukuoka 819-0395, Japan; pokharel@ed.kyushu-u.ac.jp

**Keywords:** complementary split ring resonators, metamaterials, hexagonal shape, microwave sensors, dielectric properties, Debye model, non-invasive glucose monitoring, AD8302-EVALZ board, accuracy, confidence interval

## Abstract

Accurate, non-invasive glucose monitoring remains a major challenge in biomedical sensing. We present a high-sensitivity planar microwave biosensor that progresses from a 2-cell hexagonal array to an 8-cell hexagonal array, and finally to a 16-cell double-honeycomb (DHC-CSRR) architecture to enhance field confinement and resonance strength. Full-wave simulations using Debye-modeled glucose phantoms demonstrate that the optimized 16-cell array on a Rogers RO3210 substrate substantially increases the electric field intensity and transmission response |S_21_| sensitivity compared with FR-4 and previous multi-CSRR designs. In vitro measurements using pharmacy-grade glucose solutions (5–25%) and saline mixtures with added glucose, delivered through an acrylic channel aligned to the sensing region, confirm the simulated trends. In vivo, vector network analyzer (VNA) tests were conducted on four human subjects (60–150 mg/dL), comparing single- and dual-finger placements. The FR-4 substrate ($\varepsilon_{r}$ = 4.4) provided higher frequency sensitivity (2.005 MHz/(mg/dL)), whereas the Rogers RO3210 substrate ($\varepsilon_{r}$ = 10.2) achieved greater amplitude sensitivity (9.35 × 10^−2^ dB/(mg/dL)); dual-finger contact outperformed single-finger placement for both substrates. Repeated intra-day VNA measurements yielded narrow 95% confidence intervals on |S_21_|, with an overall uncertainty of approximately ±0.5 dB across the tested glucose levels. Motivated by the larger |S_21_| response on Rogers, we adopted amplitude resolution as the primary metric and built a compact prototype using the AD8302-EVALZ with a custom 3D-printed enclosure to enhance measurement precision. In a cohort of 31 participants, capillary blood glucose was obtained using a commercial glucometer, after which two fingers were placed on the sensing region; quadratic voltage-to-glucose calibration yielded R^2^ = 0.980, root–mean–square error (RMSE) = 2.316 mg/dL, overall accuracy = 97.833%, and local sensitivity = 1.099 mg/dL per mV, with anthropometric variables (weight, height, age) showing no meaningful correlation. Clarke Error Grid Analysis placed 100% of paired measurements in Zone A, indicating clinically acceptable agreement with the reference meter. Benchmarking against commercial continuous glucose monitoring systems highlights substrate selection as a dominant lever for amplitude sensitivity and positions the proposed fully non-invasive, consumable-free architecture as a promising route toward portable RF-based glucose monitors, while underscoring the need for larger cohorts, implementation on flexible biocompatible substrates, and future regulatory pathways.

## 1. Introduction

Diabetes mellitus is a rapidly increasing chronic disease that affects millions worldwide, posing significant medical and economic burdens on healthcare systems [1,2]. It is a major contributor to early mortality and remains a critical public health challenge. The condition is characterized by impaired regulation of blood glucose levels (BGL), with normal physiological values ranging from 70 to 140 mg/dL and persistent hyperglycemia often exceeding 200 mg/dL after meals. According to global statistics from the International Diabetes Federation (2021), it indicates that over 537 million individuals are currently living with diabetes, and this figure is projected to escalate to approximately 783 million by the year 2045. The Middle East region recorded 73 million diabetic adults in 2021, and it is anticipated to reach 136 million by 2045, representing an increase of 87% [3]. Type 2 diabetes (T2D) is the most prevalent form, resulting from insulin resistance where cells fail to respond effectively to insulin, leading to impaired glucose regulation. However, T2D is often manageable with lifestyle changes and medication.

Methods for measuring BGL can be broadly categorized into invasive, minimally invasive, and non-invasive [4]. Commercial blood glucose monitoring typically relies on invasive devices, including the Omron device (Omron Healthcare Co., Ltd., Kyoto, Japan) and FreeStyle blood glucose meter (Abbott Diabetes Care Inc., Alameda, CA, USA), which require frequent fingertip punctures to obtain blood samples. While accurate and widely used, these methods cause pain and increase infection risks due to repeated skin breaches. While minimally invasive (MI) continuous glucose monitoring (CGM) systems, such as Dexcom G6/G7 (Dexcom, Inc., San Diego, CA, USA), and FreeStyle Libre (Abbott Diabetes Care Inc., Alameda, CA, USA), track glucose in interstitial fluid (ISF), they require implanted sensors under the skin for up to two weeks. These systems may cause discomfort due to the insertion process, present risks of infection or skin irritation, and require device calibration [5], whereas factors such as water exposure or physical activity can affect device adherence. These limitations drive the urgent need for accurate, cost-effective, and fully non-invasive (NI) alternatives to existing technologies [6,7]. Various NI sensor-based methodologies are currently under investigation and broadly categorized into three main approaches: electrochemical sensors [8,9], optical spectroscopy-based sensors [10,11], and electromagnetic sensing devices [12,13]. Each technique offers distinct benefits and limitations, and ongoing research seeks to develop optimal solutions that balance comfort, accuracy, and affordability for personalized CGM.

Microwave and radio frequency (RF) sensing have gained recognition as an effective NI technique for tracing BGL, offering distinct advantages over optical and electrochemical methods. RF and microwave-based glucose sensing leverages the ability of electromagnetic waves, spanning from 300 MHz to 300 GHz, to penetrate biological tissues more effectively than optical signals, particularly at lower frequencies [14]. This enhanced penetration is attributed to their low photon energy and reduced scattering, enabling interaction with subcutaneous blood-rich regions where glucose-induced dielectric changes occur. The detection principle relies on monitoring glucose-induced variations in complex permittivity, which manifest as shifts in the resonance frequency [15], alterations in the transmission coefficient (S_21_) [16,17], and changes in phase response [18]. Performance metrics such as insertion and return loss, alongside the quality factor (QF), are used to evaluate sensitivity, with a higher QF indicating stronger field confinement and sharper resonance, improving detection of subtle permittivity changes caused by glucose fluctuations [19].

Microwave sensors can be applied to anatomical sites such as fingertips, forearms, legs, and earlobes. Skin thickness and tissue composition variations affect wave penetration and glucose detection accuracy. Optimal sites with deeper wave penetration and rich blood perfusion improve measurement reliability. For instance, ear placement requires flexible, miniaturized designs to accommodate curvature and minimize signal distortion [20]. A study in [21] introduced a sock-integrated antenna that demonstrated 99.01% clinical accuracy involving many human subjects, with similar performance observed in animal models, including rats. Additionally, a dual-antenna system operating in the millimeter-wave range, applied to the human arm, achieved glucose sensitivity as low as 1.33 mmol/L [22]. The fingertip is a favorable and commonly used site for glucose monitoring, due to its ease of contact with the sensor, rich blood supply, and thin skin layer. These characteristics enhance interaction with BGL changes and dielectric properties, allowing reliable detection through resonant frequency or amplitude shifts, and this concept is adopted in the design of the proposed sensor.

Metamaterial-inspired structures [23] such as Split Ring Resonators, SRRs, and their complementary CSRRs have gained significant attention recently due to their compact design, which is less than one-tenth of the guided wavelength (<λg/10). These structures enable precise detection of subtle changes in dielectric properties, enhancing sensor performance. As a result, their integration has shown promising results in biomedical applications. For instance, M. Baghelani et al. in [24] developed a chipless, wearable SRR-based sensor operating at 4 GHz, and demonstrated sensitivity of 38 kHz/mM with a detection range of 2 to 25 mM/L (36–450 mg/dL) using a 200 μL sample directly interfaced with ISF.

CSRRs have emerged as powerful architecture offering enhanced sensitivity and field confinement, unlike SRRs. Ebrahimi et al. [25] designed a microwave microfluidic biosensor with a CSRR-coupled microstrip transmission line, where glucose samples introduced into a microfluidic channel aligned with the sensor’s active region produced measurable frequency shifts at 2.48 GHz and a sensitivity of approximately 0.5 dB/(mg/mL). Further innovations introduced a multi-parallel CSRR (MP-CSRR) sensor, comprising a 2 × 3 matrix with six resonators arranged in parallel circuits [26]. The MP-CSRR sensor demonstrated significant sensing to glucose concentration changes, achieving a frequency shift of 9.59 × 10^−2^ MHz/(mg/dL) and an S_21_ amplitude change of 7.47 × 10^−3^ dB/(mg/dL) during experimental tests, confirming the potential of multi-CSRR configurations for precise glucose monitoring.

**Figure 1:**
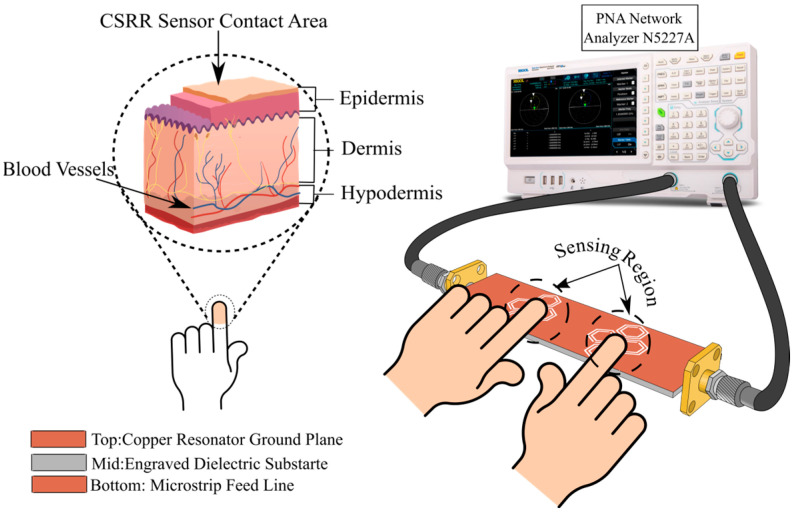
Setup of the proposed microwave sensor for continuous glucose monitoring.

In this study, we design and validate a planar, NI microwave resonator based on CSRRs for blood glucose detection. The sensor geometry progresses from a single dual-intersected hexagonal unit to an eight-cell honeycomb structure, and finally to a double-honeycomb array comprising sixteen hexagonal DHC-CSRR. Implementations on both FR-4 and Rogers substrates are explored to evaluate material effects and optimize sensitivity under single- and dual-finger contact conditions. The core objective is to detect small changes in blood glucose concentration by tracking variations in the S_21_ amplitude characteristics of the DHC-CSRR structure. A visual representation of the complete sensing system is provided in Figure 1.

The paper is organized as follows: Section 2 analyzes the dielectric properties of glucose solutions via the Debye model. Section 3 describes the CSRR sensor design, focusing on substrate selection, geometry, and performance. Section 4 presents simulation results comparing configurations. Section 5 covers fabrication, in vitro, and in vivo testing. Section 6 concludes and outlines future work toward clinical and portable applications.

## 2. Glucose-Dependent Dielectric Properties

To simulate the electromagnetic behavior of biological tissues, aqueous glucose solutions are commonly utilized in RF-based investigations due to their well-characterized dielectric properties. These aqueous glucose solutions act as blood-mimicking phantoms and exhibit dispersive behavior when subjected to electromagnetic waves across varying frequencies. Their dielectric response is frequency-dependent and can be accurately modeled using the first-order Debye relaxation model [27,28], which accounts for the polarization dynamics of dipolar molecules in the medium. The complex relative permittivity is defined as

(1) $$\varepsilon_{r}\left(\omega ,\chi \right)=\varepsilon_{r}^{\prime }\left(\omega ,\chi \right)-j\;\varepsilon_{r}^{\prime \prime }\left(\omega ,\chi \right)\;$$

where $\varepsilon_{r}^{\prime }$ and $\varepsilon_{r}^{\prime \prime }$ represent the real (energy storage) and imaginary (energy dissipation) components of the complex permittivity, respectively. A single-pole Debye model further expresses the complex permittivity as a function of angular frequency ω= 2πf and glucose concentration (*χ*), given by

(2) $$\epsilon_{r}\left(\omega ,\chi \right)=\epsilon_{\infty }\left(\chi \right)+\frac{\epsilon_{stat}\left(\chi \right)-\epsilon_{\infty }\left(\chi \right)}{1+j\omega \tau \left(\chi \right)}$$

In this formulation, the parameters $\epsilon_{stat},\;\epsilon_{\infty \;}$ denote the static and high-frequency limits of permittivity, respectively, while τ is the relaxation time.

Hofmann et al. [29,30] measured the permittivity of watery solutions up to 40 GHz using a coaxial probe with a vector network analyzer (VNA) and fitted the Debye model in which the concentration dependence of the parameters ($\epsilon_{\infty },\;\epsilon_{stat},\tau$) was described by first-order polynomials reported in Table 1.

Using the dispersion model, we computed the dielectric constant $\varepsilon_{r}^{\prime }$ and loss tangent (tan δ = $\varepsilon_{r}^{\prime \prime }/\varepsilon_{r}^{\prime })\;$at six glucose levels relevant to this study, C_1_–C_6_, corresponding to 40, 70, 100, 200, 500, and 1000 mg/dL, respectively; the values are listed in Table 2. Across this range, $\varepsilon_{r}^{\prime }$ increases slightly from 78.275 to 78.949 (+0.861%), whereas tan δ declines from 0.133 to 0.082 (−38.350%). The fractional change in tanδ is therefore much larger than the corresponding change in $\varepsilon_{r}^{\prime }$, indicating that conductivity-related losses are substantially more sensitive to glucose concentration than the real part of permittivity. Consequently, the sensor should emphasize strong field confinement and high QF resonances to produce steep transmission features capable of resolving small changes in complex permittivity.

## 3. Proposed Sensor Design

After analyzing the dielectric properties of glucose solutions in Section 2, this section outlines the process of optimizing sensor performance for a high QF resonator. The optimization begins with a basic structure comprising two hexagonal CSRR cells positioned at the center of the resonator. The structure focuses on several design parameters, including the diagonal lengths of the outer and inner cells ($D_{\mathrm{out}}$ and $D_{\mathrm{in}}$), the side width, the split gap width, the slot coupling distance (T), and the chosen substrate. To assess the impact of material properties on the sensor’s response, simulations are performed using both Rogers RT/Duroid 3210 and FR-4 substrates. Rogers, with its higher dielectric constant (10.2) and lower loss tangent (0.0027), is expected to deliver a higher QF. In contrast, FR-4 offers a lower-cost option with a dielectric constant of 4.4 and a higher loss tangent of 0.025. Both substrates are modeled with adjusted dimensions to account for their different dielectric properties. The Rogers substrate thickness is 1.27 mm, whereas FR-4 is 0.88 mm. The 50 Ω microstrip line (MTL) width, optimized in CST Microwave Studio, is 1.125 mm for Rogers and 1.5 mm for FR-4. Geometric dimensions for both designs, operating at the resonant frequency $f_{o}$ = 3 GHz, are detailed in Table 3.

**Figure 2:**
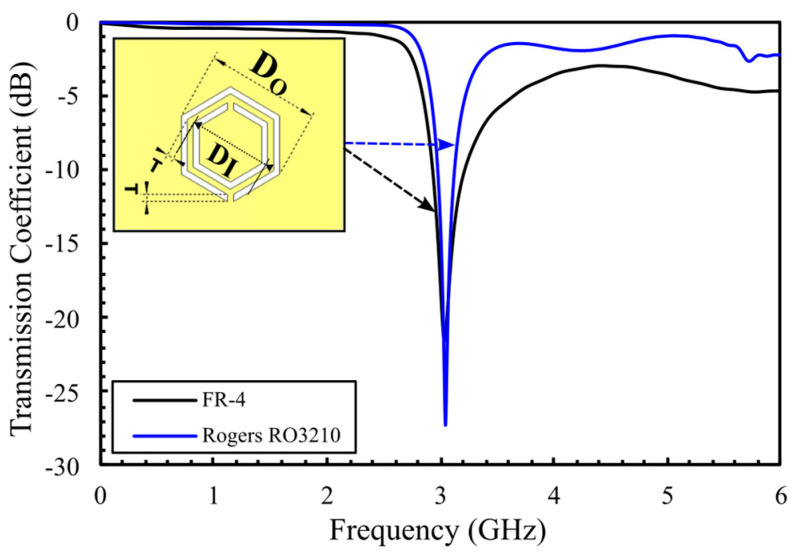
Comparison of S_21_ response between Rogers and FR-4 structures.

Figure 2 shows a comparative analysis of S_21_ response over 0–6 GHz range. The simulated results indicate that the Rogers substrate (blue curve) provides a sharper resonance, lower insertion loss, and narrower bandwidth, yielding a high QF of 218. In contrast, the FR-4 substrate (black curve) exhibits a broader resonance with a 70 MHz bandwidth and a lower QF of 43. These results highlight the superior resonance resolution and higher sensitivity achieved by Rogers, which is crucial for detecting small glucose-induced dielectric variations. The quality factor is calculated using Equation (3) in [31,32]:

(3) $$Q=\frac{f_{o}}{∆f_{3dB}}$$

where $f_{o}$ is the resonant frequency and $∆f_{3dB}\;$the bandwidth denotes it at a −3 dB level.

While numerous studies have examined geometric effects such as hexagonal [33], square [34], cylindrical [35], ring [36], and triangle [37], the main objective here is to isolate how the substrate influences sensor sensitivity rather than to propose a new structure. Compared with square and ring structures, a hexagonal lattice distributes edge fields more uniformly and increases the effective edge length, thereby confining the electromagnetic energy within the sensing region. Prior concentric-circular arrays report peak electric-field of ≈54 and 90 dB(V/m) in [26,38], and square structures report ≈ 75 and 82.5 dB(V/m) [34,39], whereas both our optimized hexagonal array and the previous study [40] attain ≈ 100 dB(V/m) (details below). The higher peak and tighter localization translate into greater ∣S_21_∣ sensitivity to small permittivity perturbations; Collectively, these results justify adopting the hexagonal geometry in our substrate–sensitivity investigation.

**Figure 3:**
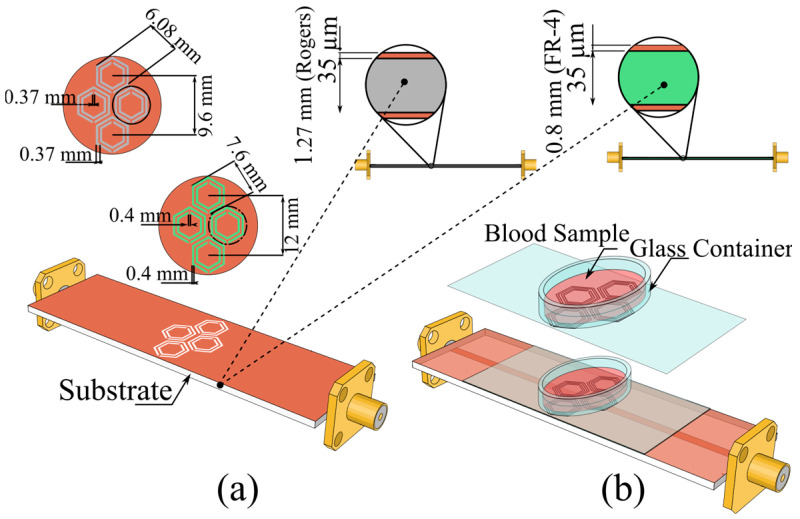
(**a**) Top view of the 8-cell hexagonal CSRR configuration on the ground plane, with detailed dimensions for Rogers and FR-4 substrates. (**b**) Schematic representation of the simulation setup featuring the integrated vein-mimicking channel.

Accordingly, we adopt the hexagonal configuration inspired by A.E. Omer et al. [33] on FR-4, re-implement it on a Rogers substrate, and optimize the design. By holding the resonator topology and operating conditions constant, this approach enables a direct, controlled comparison of substrate-dependent sensitivity. We then expanded the array from two to eight hexagonal cells and refined the dimensions, deepening the ∣S_21_∣ notch from −27.5 dB to −35 dB at $f_{o}=3$ GHz. Dimensional specifications for the optimized eight-cell layouts on Rogers and FR-4 are provided in Figure 3a.

**Figure 4:**
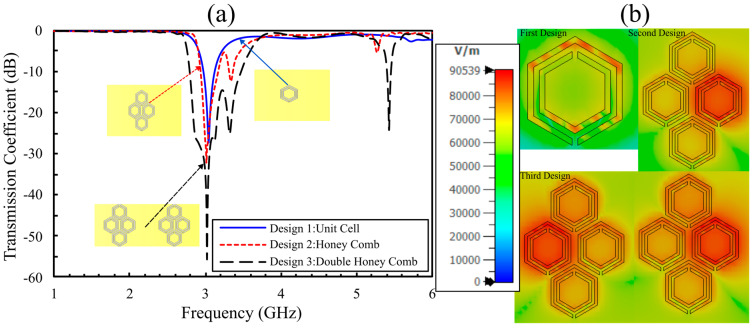
(**a**) S_21_ Simulated S_21_ comparison through different design stages. (**b**) Electric-field distribution (V/m) on the CSRR surface at the resonance frequency.

As the final optimization step, the design employs a double-honeycomb array of sixteen hexagonal cells etched into the bottom copper layer of a Rogers substrate. This configuration maximizes sensitivity, achieving a transmission coefficient of −55 dB, the highest response among all evaluated designs (Figure 4a). While Figure 4b maps the electric-field magnitude across the three hexagonal designs, showing progressive enhancement and redistribution from the single hexagonal cell (first design) to the coupled honey-cell arrangement (second), and finally to the fully optimized multi-cell configuration (third). In all cases, the field concentrates along the dielectric slits and inter-ring gaps, which define the sensing region where samples couple most strongly to the resonant near field. Quantitatively, the peak intensity increases from ~90.3 dB(V/m) (first) to ~98.2 dB(V/m) (second), reaching ~100 dB(V/m) (third). This stepwise increase in localization and peak amplitude indicates stronger capacitive coupling and higher stored energy in the resonant aperture, yielding a deeper, narrower stopband filter and greater sensitivity of the S-parameters to small permittivity changes. These attributes motivate selecting the third design as the preferred sensing platform for detecting subtle variations in glucose concentrations.

**Figure 5:**
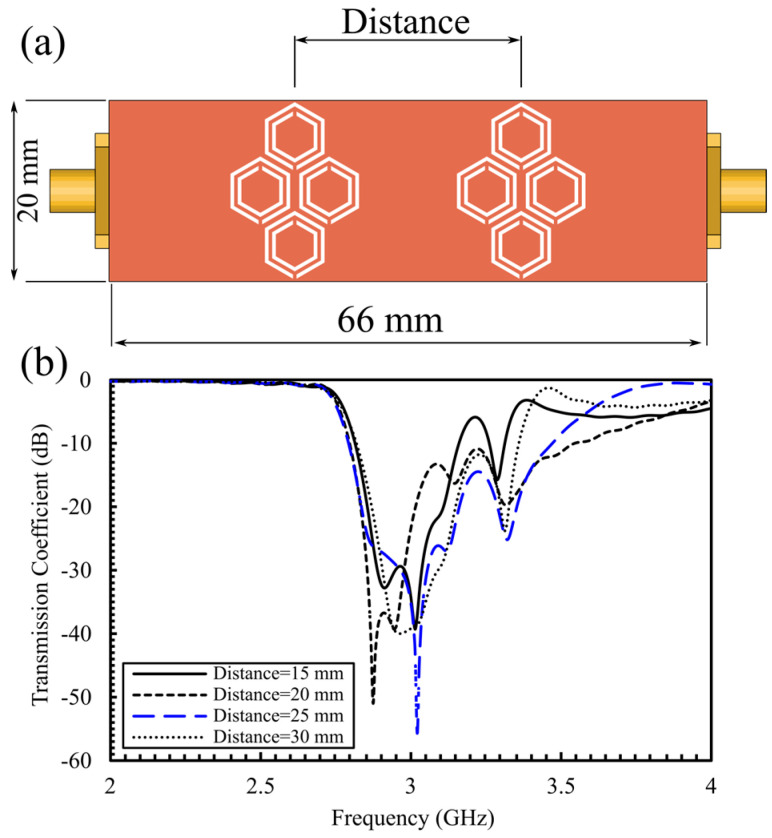
(**a**) Top view of the sensor incorporating a double-honeycomb array of 16 hexagonal CSRR sensors, (**b**) Simulated S_21_ for varying center-to-center distances (15, 20, 25, and 30 mm) between the double-honeycomb clusters.

We evaluated the center-to-center spacing between the two honeycomb clusters (15, 20, 25, and 30 mm), shown in Figure 5a. The corresponding transmission responses for these separations are shown in Figure 5b; results demonstrate that increasing separation between the honeycomb cells attenuates electromagnetic mutual coupling among neighboring hexagonal cells, leading to deeper transmission magnitudes and a higher QF. Among the tested spaces, 25 mm spacing provides the best performance, with a notch of −55 dB at 3 GHz. This spacing is selected for the final design used in simulations and experiments and is compatible with two-finger placement in the in vivo tests.

## 4. Simulation Results for Aqueous Glucose Concentrations: Comparison Between Eight- and Sixteen-Cell Hexagonal CSRR Configurations

We initiated the simulation using an eight-cell hexagonal CSRR configuration, forming a centralized honeycomb arrangement at the core of the sensor. The structure is implemented on Rogers substrate, with the CSRRs etched onto a 0.035 mm copper layer and supported by a 1.27 mm thick gray substrate, as illustrated in Figure 3b. To emulate glucose detection, an elliptical glass container filled with glucose aqueous solutions is positioned above the sensing region to mimic the presence of blood vessels. The container features a major diameter of 20 mm, a minor diameter of 14 mm, a height of 3 mm, and a wall thickness of 0.4 mm. The sample height is maintained at 1.5 mm throughout the simulation, and a 0.1 mm thick insulating film is positioned between the sensor and the container to prevent direct electrical coupling.

### 4.1. Simulation Results for the 8-Cell Single-Honeycomb Configuration

**Figure 6:**
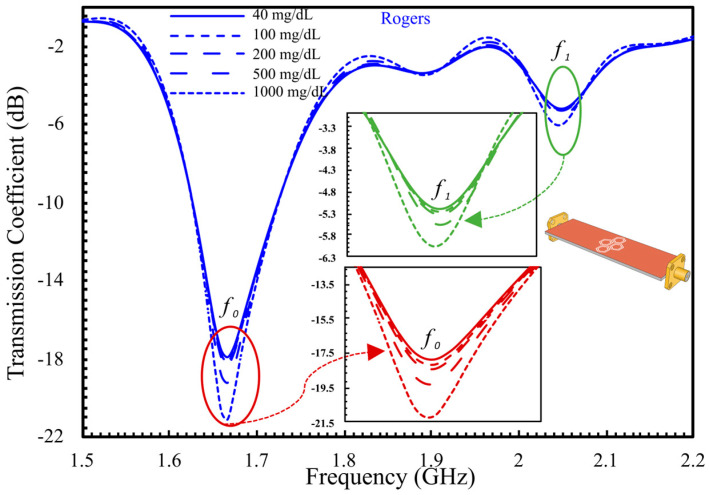
S_21_ response for different GCs with a single-honey cell CSRR.

The sensor’s performance was evaluated across a broad range of glucose concentrations: 40, 100, 200, 500, and 1000 mg/dL. Figure 6 shows the transmission response, revealing a distinct downward shift in the unloaded resonance frequency from 3 GHz to approximately 1.66 GHz ($f_{o}$) upon loading with glucose samples. The S_21_ magnitude decreases progressively with increasing glucose concentration, measuring −17.83 dB at 40 mg/dL, −18.11 dB at 100 mg/dL, −19.26 dB at 500 mg/dL, and −21.27 dB at 1000 mg/dL, corresponding to a total amplitude variation of 3.5 dB. The second harmonic ($f_{1}$) exhibits only a minor amplitude shift of 0.84 dB across the same concentration range, reflecting limited sensitivity at higher frequencies. Therefore, subsequent analysis focuses on the fundamental mode ($f_{o}$) as the dominant sensing frequency. The amplitude sensitivity, defined as $∆S_{21}/∆C$, is approximately 3.69 × 10^−3^ dB/(mg/dL), confirming the sensor’s capability to resolve glucose-induced dielectric variations and establishing a solid basis for further structural optimization. The following phase introduces a double-honeycomb configuration designed to enable dual-finger interaction, thereby enhancing sensitivity and expanding the effective sensing area.

### 4.2. Simulation Results for the Optimized 16-Cell Double-Honeycomb Configuration and Sensitivity Comparison at 15 mm and 25 mm Spacing

**Figure 7:**
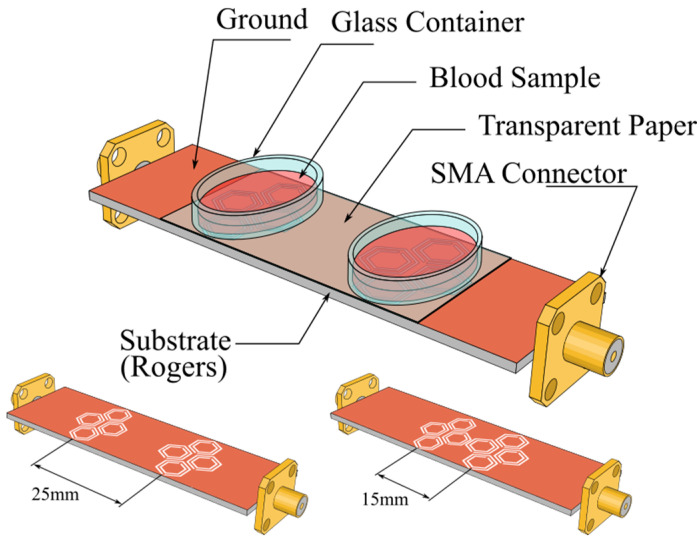
Top view of the simulated setup, illustrating the integrated sensor with two sample containers positioned over the sensing regions at center-to-center spacings of 15 mm and 25 mm.

**Figure 8:**
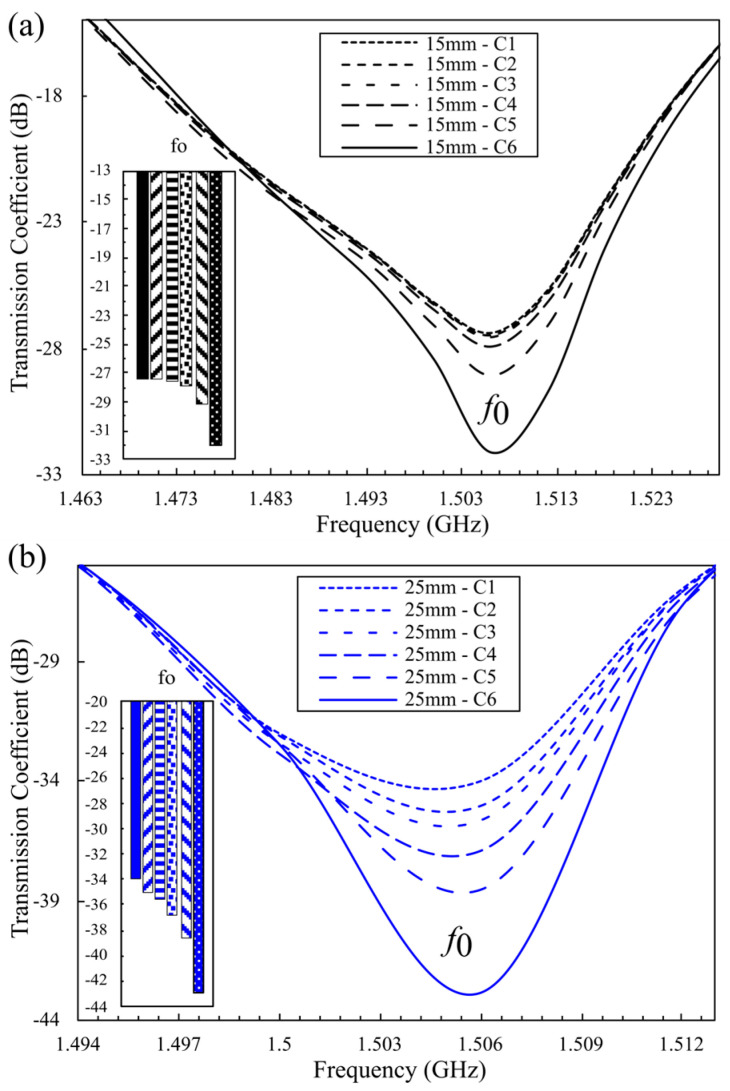
S_21_ response (amplitude) for various GC using a double-honey cell CSRR with 15 mm (black), and 25 mm (blue) center-to-center distances. (**a**) S_21_ response (amplitude) for various GC using a double-honey cell CSRR with 15 mm (black) center-to-center distances. (**b**) S_21_ response (amplitude) for various GC using a double-honey cell CSRR with 25 mm (blue) center-to-center distances.

To assess the sensitivity of the optimized sensor, we simulated six glucose concentrations (0.40, 0.70, 1.00, 2.00, 5.00, and 10.00 mg/mL; C_1_–C_6_). The biosensor comprises sixteen hexagonal CSRR cells, arranged as two clusters of eight, symmetrically distributed along the transmission line, as illustrated in Figure 7. The simulation setup comprised two elliptical plexiglass containers ($\varepsilon_{r}$ = 3.6), filled with aqueous glucose solutions, and positioned above the sensing region. This stage isolates the effect of inter-cluster spacing by comparing center-to-center distances of 15 mm and 25 mm. In both configurations, the loaded fundamental resonance appears near 1.506 GHz ($f_{o}$); however, the amplitude response differs significantly. At low concentrations (0.40–0.70 mg/mL), the 15 mm configuration exhibits a minimal change in Δ|S_21_| of 0.09 dB (−27.37 dB to −27.46 dB), as depicted in Figure 8a, whereas the 25 mm spacing shows a larger shift of 1.03 dB (−34.00 dB to −35.03 dB), shown in Figure 8b.

Herein, the 15 mm configuration produces an overall $\Delta \left|S_{21}\right|$ of 4.72 dB, while the 25 mm spacing achieves a broader variation of 8.85 dB. This corresponds to an 87.5% increase in sensitivity and a 152.86% improvement over the earlier 8-cell honeycomb design. The simulation sensitivity for the 25 mm design is approximately 3.45 × 10^−2^ dB/(mg/dL), which is 4.62 times higher than the sensitivity reported for the 2 × 3 matrix configuration [26] and 3.77 times higher than that of the triple-pole sensor [41]. These results confirm that the optimized 16-cell hexagonal CSRR configuration with 25 mm spacing provides enhanced sensitivity and measurement precision for non-invasive glucose detection. Amplitude resolution was quantified as the change in transmission magnitude per unit change in relative permittivity and loss tangent, defined as

(4) $$R_{\varepsilon }=\frac{\Delta \left|S_{21}\right|}{\Delta \varepsilon_{r}},\;R_{\tan\delta }=\frac{\Delta \left|S_{21}\right|}{\Delta \tan\delta }$$

Across the 0.40–10.00 mg/mL range, the 15 mm spacing yielded $R_{\varepsilon }=7\;dB$ and $R_{\tan\delta }=92.55\;dB$, whereas the 25 mm spacing achieved $R_{\varepsilon }=13\;dB$ and $R_{\tan\delta }=173.53\;dB$, indicating that lossy aqueous glucose solutions are tracked precisely with higher amplitude resolution at the wider spacing.

## 5. Experimental Measurements

**Figure 9:**
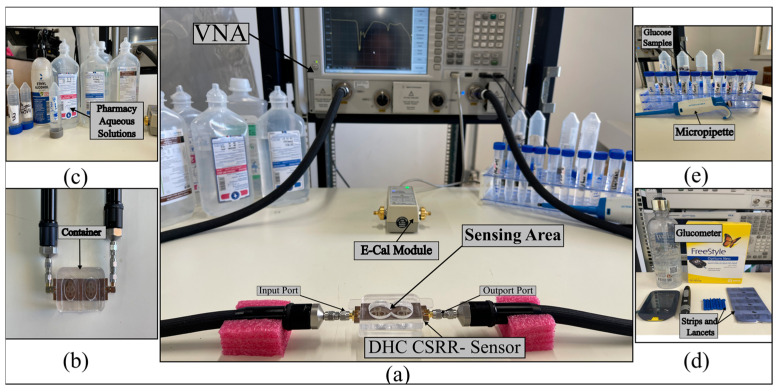
(**a**) Experimental setup for the DHC-CSRR sensor; (**b**) sensor with fluidic container for sample loading; (**c**) pharmacy-grade glucose solutions for testing; (**d**) commercial glucometer and test strips for reference measurements; and (**e**) prepared glucose samples.

Building on the promising simulation results, we performed experimental validation to assess the sensor’s performance under practical conditions. Two sensor prototypes implementing a double-honeycomb (DHC-CSRR) structure were fabricated on FR-4 and Rogers laminates with double-sided 35 µm copper cladding, using a high-resolution LPKF laser-micromachining. The experimental setup, as illustrated in Figure 9, interfaced the sensor to a Keysight PNA vector network analyzer (model N5227A, Keysight Technologies, Santa Rosa, CA, USA) via coaxial cables and employed an E-Cal module for calibration. An acrylic fluidic container was positioned above the sensing area to load liquid samples, including glucose solutions, with precise volumetric control using a micropipette. Care was taken to flush and rinse the container thoroughly with distilled water between tests and to prevent air bubble formation during loading, ensuring reliable electromagnetic measurements.

**Figure 10:**
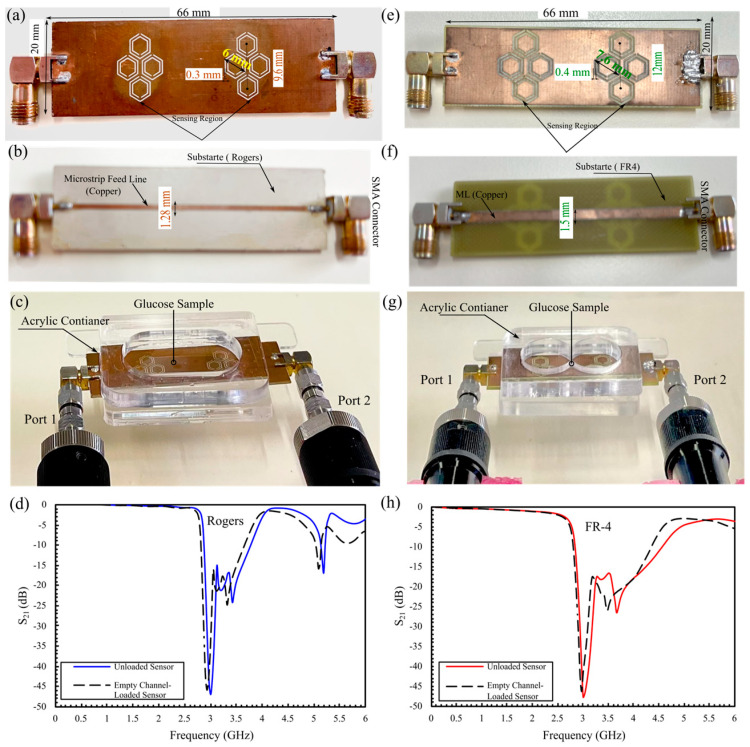
Fabricated DHC-CSRR sensors on Rogers (**a**–**d**) and FR-4 (**e**–**h**) substrates: top and bottom views; assembled setups with glucose sample; and S_21_ response comparisons.

Two DHC-CSRR sensors were fabricated for comparative testing, one on a Rogers 3210 substrate and the other on a standard FR-4, to evaluate the influence of substrate material on resonant frequency and amplitude characteristics. The top and bottom views are illustrated in Figure 10a,b (Rogers) and Figure 10e,f (FR-4). Each sensor was integrated with an acrylic fluidic container positioned directly above the sensing region, enabling the channel to be loaded with liquid test samples, as shown in Figure 10c,g. Before testing, a full calibration of the VNA was performed to eliminate systematic errors and ensure highly accurate readings. Following calibration, S-parameter measurements were carried out at the same room temperature to maintain consistent test conditions. Figure 10d,h depict the S_21_ response of both sensors before and after loading with an empty container. Both sensors initially resonated at 3.00 GHz with an amplitude of approximately −47 dB. After placing the empty container, the Rogers sensor showed a 33 MHz frequency shift and an amplitude of −45.9 dB, whereas the FR-4 sensor showed a 30 MHz frequency shift and an amplitude of −46.2 dB. These mall shifts represent the baseline dielectric loading effect of the container, providing a reference point for subsequent glucose concentration measurements.

### 5.1. S_21_-Based In Vitro Detection of Glucose in Deionized Water, and Evaluation Across Saline

Following fabrication and baseline characterization, the sensors’ performance was evaluated under varying glucose concentrations. Three commercially available dextrose solutions (5%, 10%, and 25% *w*/*v*) were tested by dispensing 320 µL of solution into the acrylic container to ensure uniform channel filling and consistent dielectric loading. To minimize environmental and instrumental influences, all measurements were conducted in a temperature-controlled laboratory at 25 ± 1 °C, with all glucose samples held at the same temperature before acquisition. Because the CSRR’s scattering response is governed by the glucose solution’s temperature-dependent permittivity, temperature control is essential. Prior work on CSRR sensors reports a small resonant-amplitude temperature coefficient (≈0.03–0.09 dB/°C) [42]; therefore, slight ambient temperature changes are not expected to materially affect the ∣S_21_∣ response.

**Figure 11:**
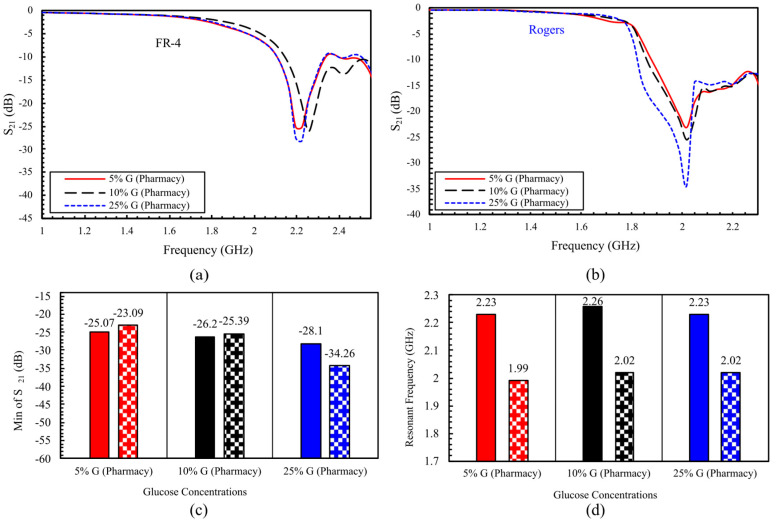
Measured transmission responses of the DHC-CSRR sensors for 5%, 10%, and 25% glucose: (**a**) FR-4 sensor; (**b**) Rogers sensor; (**c**) comparison of minimum ∣S_21_∣ (solid bars: FR-4, patterned bars: Rogers); and (**d**) resonant-frequency shifts across concentrations.

A comparative analysis of the two sensor designs revealed distinct response behaviors. The response of the loaded FR-4 sensor is shown in Figure 11a. At 5% glucose concentration, the S_21_ amplitude is −25.072 dB at 2.226 GHz. Increasing concentration to 10% yields S_21_ = −26.195 dB at 2.25625 GHz, corresponding to an amplitude change of 1.123 dB and a frequency shift of 30 MHz. At 25%, S_21_ further decreased to −28.096 dB, while the resonance returned to 2.23 GHz. Overall, across the three concentrations, the FR-4 sensor exhibits a total amplitude change of 3.024 dB and a net frequency shift of 30 MHz. By contrast, the Rogers-based sensor in Figure 11b shows a larger amplitude variation in S_21_, with a total change of 11.17 dB across the tested glucose range (Figure 11c), approximately 3.7 times greater than the change observed for the FR-4 sensor. Additionally, while the FR-4 resonance frequency increases by 10% and then returns to its initial value at 25%, the Rogers sensor maintains a stable frequency shift from 10% to 25%, as seen in Figure 11d.

**Figure 12:**
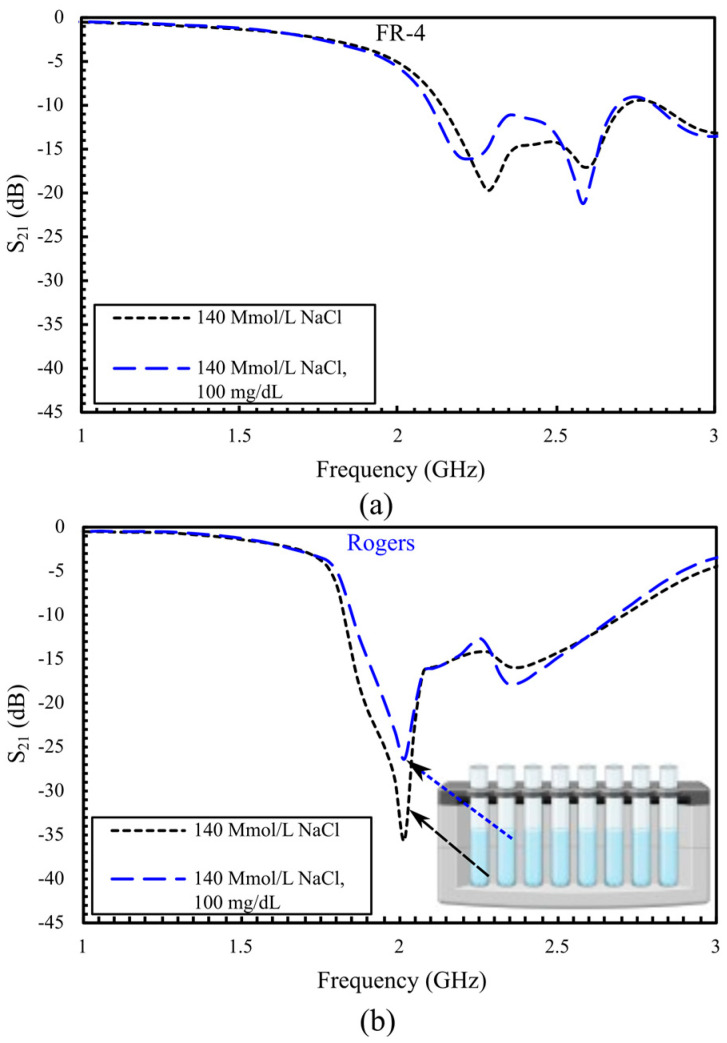
|S_21_| responses under saline condition and with added glucose: (**a**) FR-4 sensor; (**b**) Rogers sensor.

After evaluating pharmaceutical-grade glucose solutions, we conducted additional experiments using saline solutions prepared by dissolving sodium chloride (NaCl) in distilled water. Two conditions were tested: 140 mmol/L NaCl, representing sodium levels within the normal physiological range (normonatremia), and a physiological mixture of 140 mmol/L NaCl with 100 mg/dL glucose, mimicking real-world blood conditions with both sodium and glucose present, as shown in Figure 12. The S_21_ amplitude at 140 mmol/L NaCl solution is −19.7 dB with a resonant frequency of 2.286 GHz for the FR-4 structure in Figure 12a. Adding glucose reduces S_21_ to −21.32 dB and shifts the frequency to 2.196 GHz, resulting in an amplitude reduction of 1.62 dB and a frequency shift of approximately 89 MHz. Figure 12b shows the Rogers sensor response under identical conditions, with −35.579 dB at 2.016 GHz, at 140 mmol/L NaCl. Upon adding glucose, the amplitude of S_21_ increased to −26.567 dB while the frequency remained unchanged. These experimental observations accord with material properties. As Rogers RO3210 substrate has a low loss tangent (tan δ ≈ 0.0027), the transmission magnitude is more responsive to dielectric losses in samples, whereas the lower permittivity of FR-4 (ε_r_ ≈ 4.4) increases effective-permittivity contrast with the sample and thus produces larger resonance–frequency shifts.

**Figure 13:**
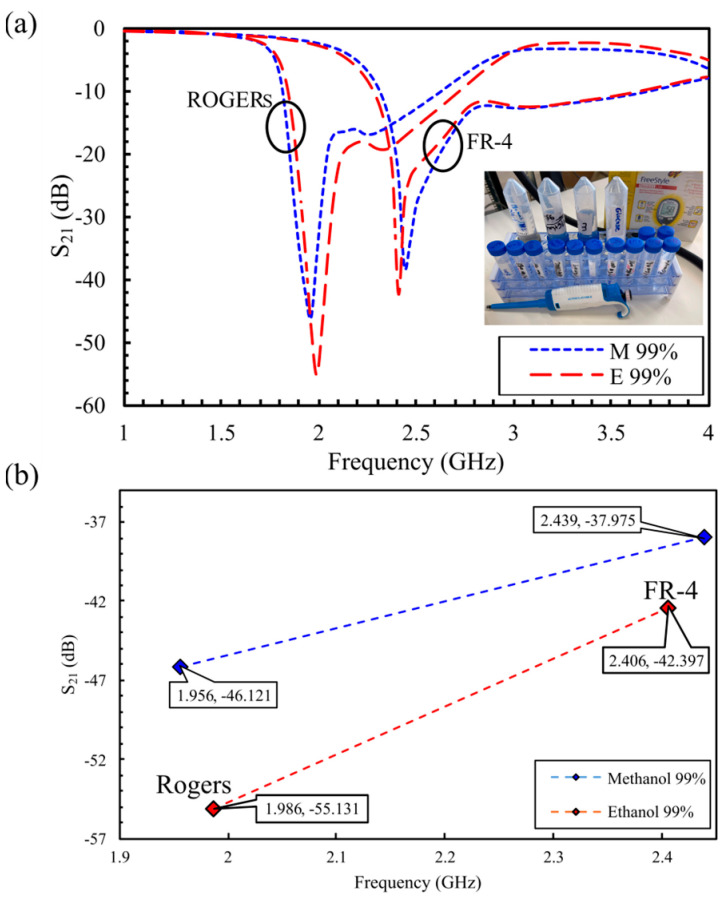
(**a**) Measured transmission coefficients for 99% methanol and ethanol on FR-4 and Rogers, and (**b**) extracted resonant frequency and amplitude for each sensor.

To extend the in vitro characterization beyond aqueous samples, both resonators were evaluated using anhydrous alcohol (99% methanol and 99% ethanol). The liquid samples are distinguished by tracking shifts in the resonant features of the S_21_ response within the 1–4 GHz band, as depicted in Figure 13a. For the Rogers structure, the amplitude contrast between methanol and ethanol is pronounced (Δ|S_21_| ≈ 9.0 dB; −46.12 dB for methanol versus −55.13 dB for ethanol at ~2.0 GHz), whereas the FR-4 sensor exhibits a more modest amplitude separation (Δ|S_21_| ≈ 4.42 dB; −37.98 dB for methanol versus −42.40 dB for ethanol at ~2.4 GHz). In terms of frequency resolution, the FR-4 response shifts downward by ~35 MHz between the two samples, while the Rogers resonance shifts by ~31 MHz (1.956 GHz to 1.9867 GHz). Figure 13b summarizes the |S_21_| attributes for each liquid sample by reporting the mean resonance frequency and amplitude within the 1.9–2.5 GHz interval. These findings are consistent with the material-dependent sensing behavior observed in the glucose and saline experiments: the Rogers implementation primarily enhances amplitude-based detection, providing a larger dynamic range in |S_21_|, and the FR-4 implementation affords clearer tracking of resonant frequency near 2.4 GHz. Overall, the two substrates, interrogated with the same CSRR-based metamaterial topology, yield distinct resonant features for different liquids, reflecting contrasts in their dielectric properties and underscoring the high sensitivity of the proposed design.

### 5.2. Comprehensive Evaluation of Glucose Sensing Performance Using FR-4 and Rogers Substrates in One- and Two-Finger Configurations

Most previous studies have not experimentally compared single- versus dual-finger placement on sensor sensitivity; instead, they typically adopt a single configuration without demonstrating both. In this section, we address this gap by systematically evaluating and comparing the sensitivity of FR-4 and Rogers substrates under one- and two-finger cases. Experimental validation was performed with four human volunteers (two males, two females), spanning distinct blood glucose levels of approximately 60, 70, 90, and 150 mg/dL for Volunteers 1–4, respectively. For each volunteer, three repeated measurements were acquired, and the reported glucose values represent the mean glucometer readings (tolerance ±3 mg/dL) rounded to the nearest multiple of 5 mg/dL. Participants were 28–52 years old, 160–185 cm in height, and 70–110 kg in weight, yielding Body Mass Index (BMI) values from 24.5 to 33.9 kg m^−2^, thus covering both normal-weight and overweight categories.

**Figure 14:**
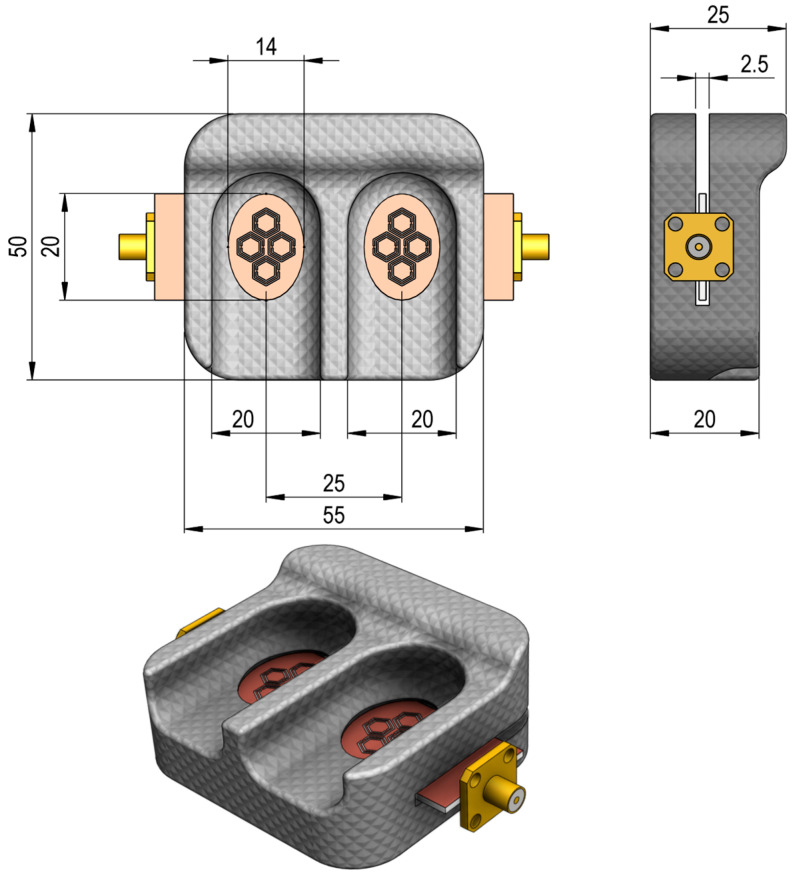
Top and side views of the sensor housing with precise dimensions optimized for stable human finger placement.

A custom 3D-printed enclosure made from PLA plastic was used to house the sensor and enhance measurement accuracy. The enclosure, as shown in Figure 14, features a dual-cavity design, each cavity 20 mm deep and 14 mm wide, to enable stable, reproducible finger placement over the sensing region. Flanges at both ends provide secure mounting, and the side view indicates a 2.5 mm separation between cavities with a total enclosure width of 55 mm. This robust enclosure minimizes measurement errors by maintaining proper alignment and consistent pressure on the sensing area, thereby ensuring reliable data collection during experiments. The measurement setup comprised a VNA connected to the sensor and enclosure via coaxial cables, and responses were recorded as S-parameters.

#### 5.2.1. In Vivo Glucose Detection in Humans Across 60–150 mg/dL (Single-Finger Configuration Using FR-4 and Rogers Substrates)

**Figure 15:**
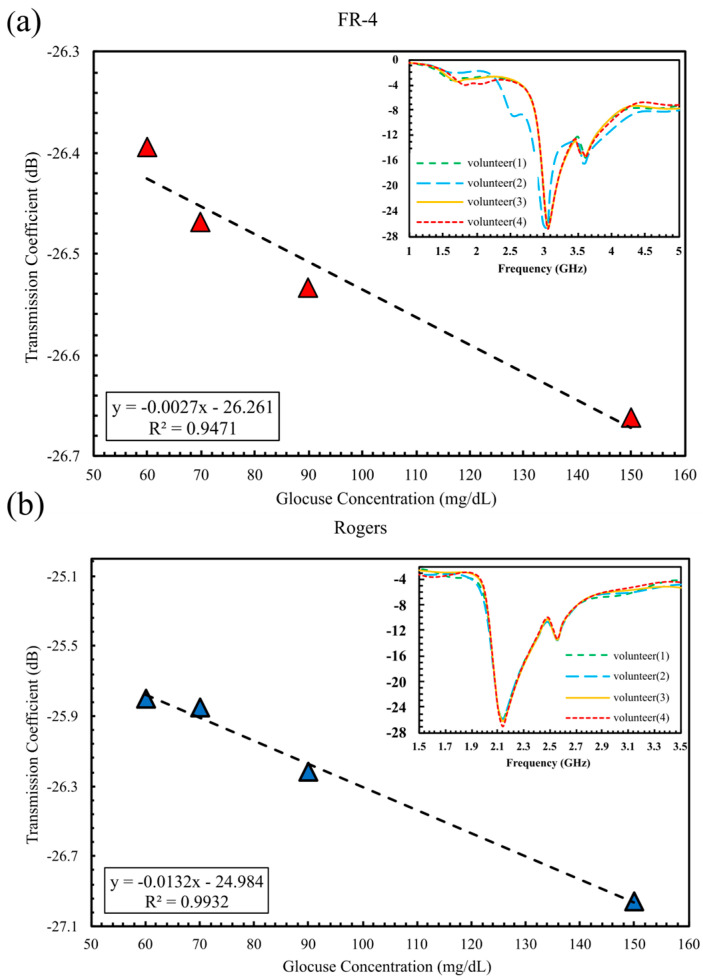
S_21_ magnitude versus glucose concentration measured noninvasively with single-finger placement on (**a**) FR-4 substrate and (**b**) Rogers substrate.

The initial in vivo experiment required each volunteer to place a single finger on the sensor to assess its response, enabling a direct comparison between FR-4 and Rogers implementations under realistic physiological conditions, as presented in Figure 15. The FR-4-based sensor shows a gradual amplitude decrease with increasing glucose. S_21_ declined from −26.39 dB at 60 mg/dL (volunteer 1) to −26.66 dB at 150 mg/dL (volunteer 4). The total S_21_ variation across this range is 0.2683 dB, corresponding to a sensitivity of 0.00754 dB/(mg/dL), with a strong linear fit, as illustrated in Figure 15a. In comparison, the Rogers-based sensor demonstrates a broader response, with S_21_ decreasing from −25.79 dB to −26.96 dB over the same glucose range in Figure 15b, corresponding to an amplitude change of 1.1601 dB and a higher sensitivity of 0.0122 dB/(mg/dL). Although both sensors correlate strongly with glucose level (R^2^ = 0.947 for FR-4; R^2^ = 0.993 for Rogers), the Rogers amplitude range is ~4.3 × larger than FR-4, while the resonance frequency remains largely unchanged for both.

#### 5.2.2. In Vivo Glucose Detection Utilizing DHC-CSRR on Rogers and FR-4 Substrates (Dual-Finger Configuration)

**Figure 16:**
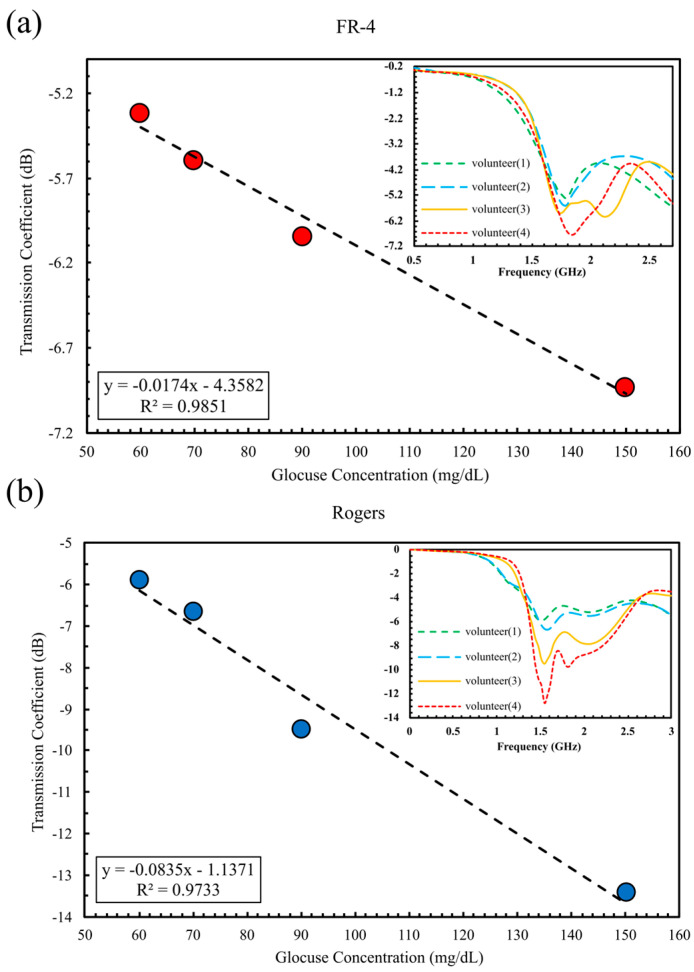
∣S_21_∣ magnitude versus glucose concentration measured with two-finger placement on (**a**) FR-4 substrate and (**b**) Rogers substrate.

Simulation results established that the sixteen-cell double-cluster configuration delivers higher sensitivity than the single-cluster design. To experimentally validate this enhancement, both FR-4 and Rogers sensors were tested under identical conditions using dual-finger placement. For the FR-4 sensor operating at 1.85 GHz, it records an S_21_ shift from −5.31 dB for volunteer 1 to −6.93 dB for volunteer 4, as depicted in Figure 16a, providing an amplitude range of 1.615 dB and a sensitivity of 0.027 dB/(mg/dL), with a nearly perfect curve fit (R^2^ = 0.985).

Additionally, the Rogers-based sensor, shown in Figure 16b, which operates at 1.51 GHz, delivers a substantially larger amplitude variation ranging from −5.89 dB to −12.71 dB, corresponding to a sensitivity of 0.0935 dB/(mg/dL). Although its curve fit is slightly lower (R^2^ = 0.973), the Rogers device demonstrated a markedly higher amplitude sensitivity, approximately 3.36 times greater than that of FR-4. In frequency shift analysis, however, the FR-4 sensor performed better with a sensitivity of 2.005 MHz/(mg/dL), compared to 3.88 × 10^−1^ MHz/(mg/dL) for the Rogers substrate. All in vivo experimental results are summarized in Table 4.

**Figure 17:**
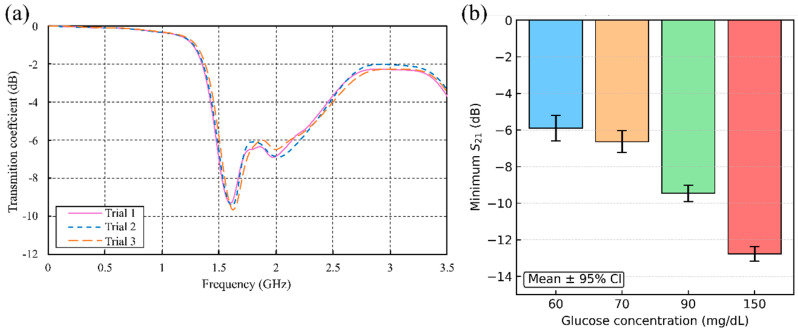
(**a**) Measured ∣S_21_∣ for a single participant at 90 mg dL^−1^ across three consecutive trials (*n* = 3), and (**b**) mean resonant ∣S_21_∣ amplitude for each tested glucose concentration (60, 70, 90, 150 mg dL^−1^); error bars denote the 95% confidence interval of the mean.

To quantify the measurement precision and repeatability of the proposed Rogers-based sensor, a comprehensive uncertainty analysis was performed. Each participant performed three measurements (n = 3), in an intra-day manner, as illustrated in Figure 17a, providing a total of 12 readings for the analysis. For each participant’s set of three replicates, the mean ($\bar{x}$) and sample standard deviation (s) of the resonant |S_21_| amplitude were calculated. Measurement uncertainty was then characterized using the 95% confidence interval (CI) [43] of the mean, defined as

(5) $$CI=\bar{x}\pm U_{95\%}\;$$

With

(6) $$U_{95\%}=t_{0.025,\nu }\cdot \left(SEM\right)=t_{0.025,\nu }\cdot \left(\frac{s}{\sqrt{n}}\right)\;$$

where $U_{95\%}$ is the expanded uncertainty at the 95% confidence level, $s/\sqrt{n}$ is the standard error of the mean (SEM), n = 3 is the number of replicates, and $t_{0.025,\nu }$ is the two-tailed Student’s t-value for a 95% confidence level with ν=n−1=2 degrees of freedom ($t_{0.025,2}$ = 4.303) [43].

The results of this analysis are summarized in Figure 17b, which plots the mean ∣S_21_∣ response for each glucose concentration with error bars denoting the 95% CI. Shorter error bars indicate higher repeatability at the corresponding glucose level. For example, the participant with a glucose level of 90 mg/dL (Volunteer 3) exhibited three replicate s_21_ measurements of −9.283, −9.443, and −9.643 dB at a resonant frequency of 1.58 GHz (Figure 17a), yielding a mean of −9.456 dB and a 95% CI of [−9.904, −9.008] dB. As reported in Table 5, the numerical analysis indicated that the lowest glucose level (60 mg/dL) exhibited the largest uncertainty (s = 0.284 dB; $U_{95\%}$ = 0.706 dB), while the 150 mg/dL concentration showed the smallest variation (s = 0.162 dB; $U_{95\%}$ = 0.401 dB). To assess overall sensor repeatability across all measurements, the pooled standard deviation ($s_{p}$) was calculated as

(7) $$s_{p}=\sqrt{\frac{\sum_{i=1}^{k}\left(n_{i}-1\right)\;s_{i}^{2}}{\sum_{i=1}^{k}\left(n_{i}-1\right)}}\;\;$$

where k=4 is the number of groups (participants). This yields $s_{p}\approx 0.22$ dB (one standard deviation, 1σ), corresponding to a pooled expanded uncertainty of approximately ±0.5 dB at a 95% confidence level. This statistical analysis supports the precision and reliability of the proposed sensor for non-invasive glucose monitoring.

The proposed sensor, implemented on a Rogers substrate with sixteen hexagonal cells, is benchmarked in Table 6 against recent state-of-the-art microwave glucose sensors, using both transmission S_21_ and reflection S_11_ responses. The reported glucose concentration ranges span from very dilute glucose levels (0–160 mg/dL) to extremely wide ranges (0–20,000 or 0–30,000 mg/dL), whereas this work targets a narrow, physiologically relevant blood glucose range of 60–150 mg/dL. Because “sensitivity” is defined as the average slope in dB/(mg/dL) over the tested range, devices characterized over broad, largely non-physiological ranges naturally exhibit smaller normalized slopes, whereas sensors optimized around normoglycemic levels show higher local sensitivities. Accordingly, the 9.4 × 10^−3^ dB/(mg/dL) reported here reflects high local amplitude sensitivity within the clinically relevant band, and direct comparison with biosensors evaluated over much wider or different ranges should be interpreted with caution. Moreover, the in vivo cohort consisted of healthy volunteers with glucose values predominantly in the euglycaemic range, where dielectric contrasts are relatively small; at higher glucose concentrations, the larger permittivity variations would be even easier to detect with the proposed high-sensitivity sensor.

#### 5.2.3. In Vivo Human Validation (N = 31) of a Dual-Finger DHC-CSRR Sensor on Rogers Using an AD8302 Evaluation Board

Following S-parameter measurements on four volunteers using a VNA, the DHC-CSRR dual-finger sensor on a Rogers substrate achieved greater changes in transmission magnitude than the FR-4 variant. Accordingly, the |S_21_| amplitude resolution was adopted as the primary sensitivity metric for subsequent human testing. The next day, 31 student volunteers (undergraduate and postgraduate; 19 males, 12 females) were enrolled for capillary blood sampling via finger-prick. Participants characteristic are reported as a mean ± standard deviation (SD): age of 22.3 ± 3.3 years (range 18–29), height 173.9 ± 9.8 cm (155–190), weight 76.6 ± 11.5 kg (52–97), BMI 25.3 ± 2.9 kg·m^−2^ (20.0–31.6; BMI = weight [kg]/height [m]^2^), and capillary glucose 90.8 ± 16.5 mg·dL^−1^ (approximate 60–125 mg·dL^−1^). Full descriptive statistics are provided in Table 7.

To ensure accurate glucose measurements, volunteers fasted for at least 1 h before testing, and all measurements were performed in a temperature-controlled laboratory at 25 ± 1 °C. Before each reading, participants washed and thoroughly dried their hands, and reference capillary blood glucose for each volunteer was obtained using a commercial FreeStyle glucometer, which served as the reference standard. To improve signal consistency, all participants followed a unified finger-placement protocol: the index and middle fingers of the right hand were gently placed on the sensing area while a custom housing was positioned over the sensor to minimize pressure variability and enforce consistent two-finger alignment. The same protocol was applied in both the VNA-based setup and the AD8302 evaluation-board setup, with comparable contact durations across volunteers, and in each case, the resulting reading was recorded promptly once the signal had stabilized.

**Figure 18:**
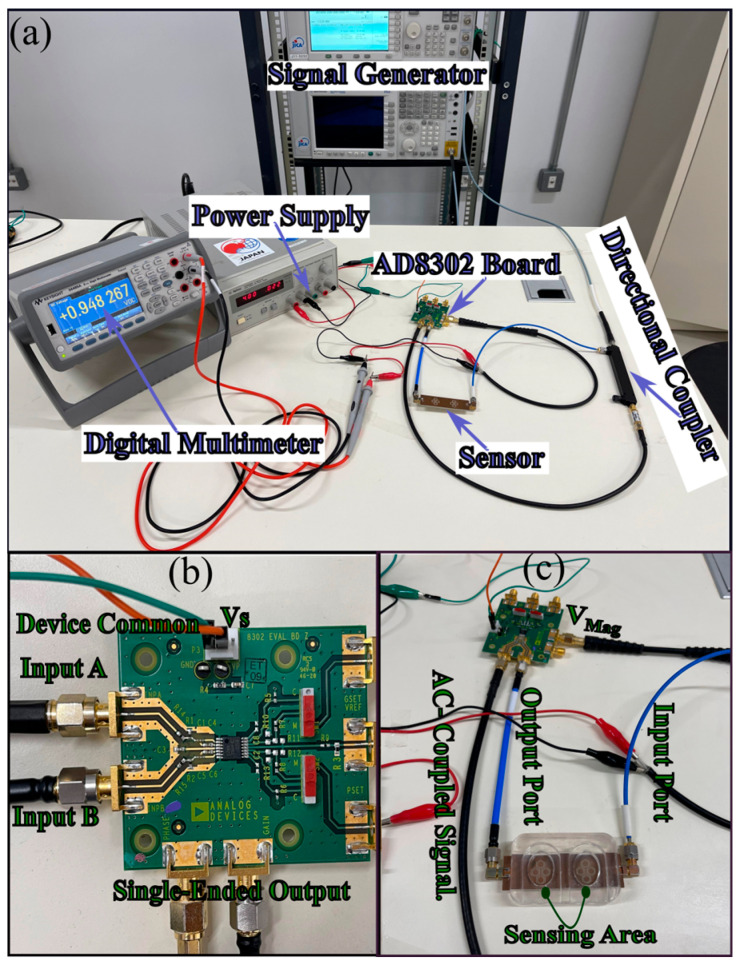
(**a**) Experimental setup for non-invasive glucose measurement comprising signal generator, directional coupler, glucose sensor, AD8302 evaluation board, DC supply, and multimeter (measuring $V_{mag}$), (**b**) AD8302-EVALZ pinout showing Inputs A/B, V_S_, ground, and the single-ended output, and (**c**) two-finger sensor enclosure with RF input/output and AC-coupled paths to V_mag_.

As a compact alternative to the VNA setup, we implemented an RF gain/loss detection setup based on the AD8302 evaluation board as demonstrated in Figure 18. The board operates from 2.7 to 5.5 V, accepts AC-coupled signal, single-ended inputs in a 50 Ω environment over −60 to 0 dBm, and supports bandwidths up to 2.7 GH, providing a DC output proportional to RF gain. The single-ended inputs were impedance-matched and connected directly to the directional coupler. The sensor under test was inserted into the RF chain and driven by a continuous-wave source at the previously identified most-sensitive operating frequency of 1.5 GHz, with an output power of −20 dBm to remain safely below the 0 dBm maximum recommended at the AD8302 inputs. As illustrated in Figure 18a, the signal generator feeds the directional coupler, whose through port drives the sensor input [Figure 18c], while the coupled port provides the reference signal to AD8302 Input A [Figure 18b]; the sensor output is routed to AD8302 Input B. The AD8302 is powered from a bench supply at 4.0 V (V_S_) with the return tied to Device Common (GND), and the magnitude output (V_MAG_) is read using a digital multimeter [Figure 18a,c]. With the bare (unloaded) sensor, the multimeter indicates approximately 950 mV. All interconnects are 50 Ω SMA coaxial cables to preserve impedance matching.

**Figure 19:**
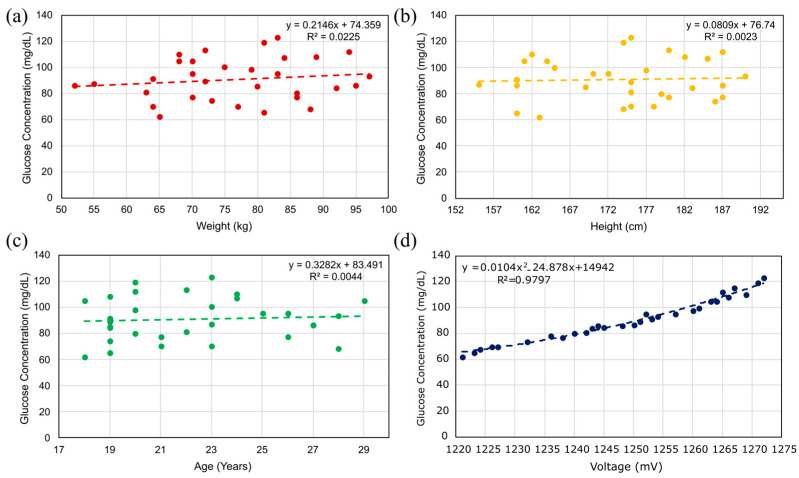
Correlation between capillary glucose and participant attributes: (**a**) weight (kg); (**b**) height (cm); (**c**) age (years); (**d**) sensor output (mV). N = 31. Least-squares fits are shown (linear in (**a**–**c**); quadratic in (**d**)).

Figure 19a–c evaluates the dependence of measured glucose concentration on body weight, height, and age, respectively, and demonstrates negligible correlation in all cases (R^2^ ≈ 0.002–0.004). This indicates that, within the studied cohort, these demographic variables do not act as secondary confounders for the sensor’s readings.

To verify the functionality of the proposed microwave sensor and the AD8302-based readout board calibrated at 1.5 GHz, we conducted an in vivo study on 31 volunteers. Each participant placed two fingers on the sensing region, and the board’s output V_MAG_ (millivolts, mV) was recorded instantaneously. As illustrated in Figure 19d, measurements clustered within the euglycemic range: 14/31 (45%) fell between 80 and 100 mg/dL (e.g., 95 mg/dL at 1257 mV; 100 mg/dL at 1261 mV), whereas only 4/31 (13%) lay in the lower tail (65–70 mg/dL), including the minimum 62 mg/dL at 1221 mV. Evaluated glucose levels were uncommon: four participants exceeded 110 mg/dL (112–123 mg/dL at 1265–1272 mV).

A second-order calibration mapping voltage to glucose was then fitted:

(8) $$G\left(V\right)=aV^{2}+bV+c$$

With coefficients a = 1.040 × 10^−2^, b = −24.878, and c = 1.494 × 10^4^. The model achieved a coefficient of determination $R^{2}=0.980$. For interpretability, the local sensitivity (mg/dL per mV) follows the analytical derivative

(9) $$\frac{dG}{dV}=2\;A\;V+B$$

which was evaluated at the dataset mean voltage $\bar{V}=\frac{1}{31}\sum_{i}V_{i}$ = 1.251 V, and the slope is ${\left.\frac{dG}{dV}\right|}_{V=\bar{V}}=1.099\mathrm{mg}\;dL^{-1}\mathrm{per}\;mV^{-1}$.

For the measurements reported here, the calibration curve in Figure 19d is valid for a fixed reference signal and AC excitation at the specified power level under stable bench conditions. Potential long-term calibration drift and sensor degradation were not assessed in this work and will require a dedicated longitudinal study, which we identify as an important direction for future work.

Model errors were quantified using standard metrics. Let $e_{i}=y_{\mathrm{pred},i}-y_{\mathrm{refr},i}$ denote the signed residual for the sample i, where $y_{\mathrm{pred}}\;$and $y_{\mathrm{refr}}\;$are the predicted and reference glucose (mg/dL), respectively, and N is the number of samples. The mean absolute error (MAE) and the root–mean–square error (RMSE) are defined below:

(10) $$\mathrm{MAE}=\frac{1}{N}\sum_{i=1}^{N}\left|e_{i}\right|$$

(11) $$\mathrm{RMSE}=\sqrt{\frac{1}{N}\sum_{i=1}^{N}{\left(e_{i}\right)}^{2}}$$

with numerical values MAE = 1.980 mg/dL and RMSE = 2.316 mg/dL. The mean absolute percentage error is MAPE = 2.167%, corresponding to an overall accuracy = 97.833%, where

(12) $$\mathrm{MAPE}=\frac{100}{N}\sum_{i=1}^{N}\frac{\left|e_{i}\right|}{Y_{\mathrm{Refr},i}}\%$$

Error dispersion is low with a standard deviation of absolute errors (SDAE) = 2.354 mg/dL, computed from

(13) $$\mathrm{SDAE}=\sqrt{\frac{1}{N-1}\sum_{i=1}^{N}{\left(e_{i}-\bar{e}\right)}^{2}},\;\bar{e}=\frac{1}{N}\sum_{i=1}^{N}e_{i}$$

Taken together, the tight alignment of observations to the quadratic fit, the modest dispersion of residuals, and the local sensitivity of $1.099\;\mathrm{mg}/\mathrm{dL}\;\mathrm{per}\;{\mathrm{mV}}^{-1}$ indicate that the AD8302-based readout provides a reliable quantitative mapping from voltage to glucose for this cohort.

**Figure 20:**
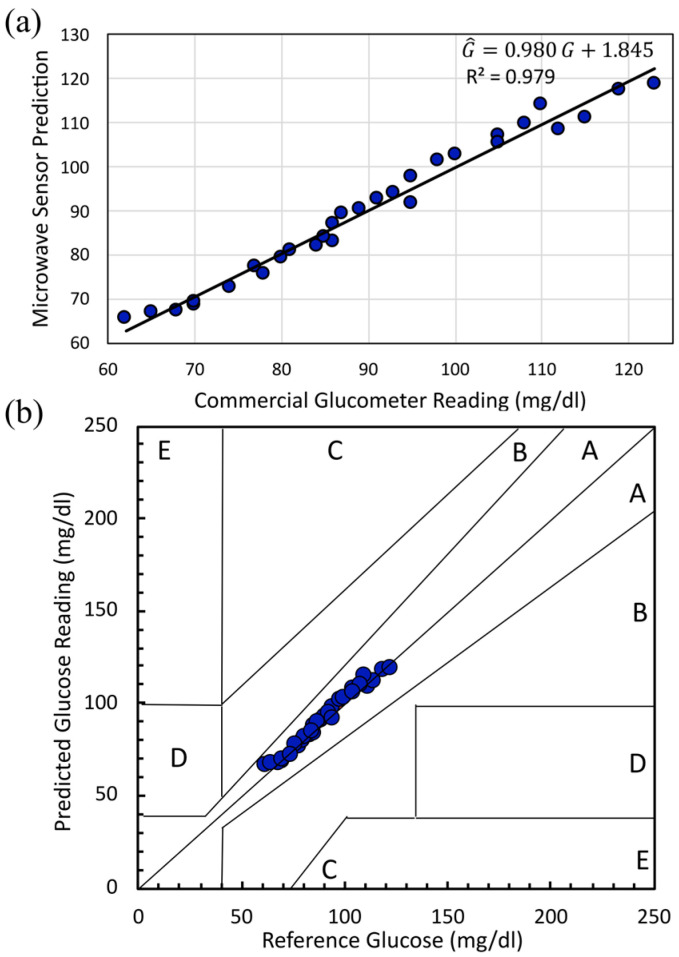
(**a**) Calibration of the microwave sensor against finger-prick reference glucose for the full human-subject dataset. (**b**) Clarke Error Grid of predicted glucose in 31 samples.

To evaluate predictive performance and clinical accuracy, we fitted an ordinary least squares (OLS) regression of predicted glucose $\left(\hat{G},\;\mathrm{mg}/\mathrm{dL}\right)\;$on reference glucose $\left(G,\;\mathrm{mg}/\mathrm{dL}\right)$, as illustrated in Figure 20a. The relationship is

(14) $$\hat{G}=m\;G+b$$

with slope m=0.980, intercept b=1.845 mg/dL (*n* = 31; reference range 62–123 mg/dL). The slope’s proximity to unity and the small positive intercept imply negligible proportional and constant bias across the observed range.

Clinical accuracy was quantified using the Clarke Error Grid Analysis (EGA), as shown in Figure 20b. Zone A denotes predictions within ±20% of the reference value, including the hypoglycemia range (G<70 mg/dL). All 31 measurements (100%) fell in Zone A, with none in Zones B–E. At the lowest boundary, the largest relative error occurs at G=62 mg/dL, for which the model predicts $\hat{G}=65.689\;$mg/dL (absolute error 3.689 mg/dL, relative error 5.951%), which remains within Zone A. Within the euglycemic range, representative cases include G=86 mg/dL with $\hat{G}=87.070\;$mg/dL (1.070 mg/dL, 1.244%), G=105 mg/dL $with\;\hat{G}=105.497\;$mg/dL (0.497 mg/dL, 0.474%), and G=115 mg/dL $with\;\hat{G}=111.202$ mg/dL (3.798 mg/dL; 3.303%). Although these Clarke Error Grid results demonstrate excellent clinical accuracy, the present in vivo dataset is confined to normoglycemic young adults, with capillary glucose values spanning approximately 60–125 mg/dL (90.8 ± 16.5 mg/dL). Within this relatively narrow interval, the sensor exhibits high amplitude sensitivity and low experimental uncertainty, enabling the resolution of small glucose differences in a healthy cohort. In practical terms, larger excursions in glucose concentration, such as those occurring in prediabetic and diabetic states, are expected to induce larger permittivity changes and therefore be easier to detect with the same system. Future studies will investigate an expanded glycemic range to confirm performance across a broader spectrum of clinically relevant glucose levels and to refine the calibration for sustained hyperglycemia.

In Table 8, we provide a concise comparison between current-generation invasive capillary meters, state-of-the-art continuous glucose monitoring systems, and the proposed fully non-invasive dual-finger DHC-CSRR sensor in terms of clinical accuracy (mean absolute relative difference, MARD, or equivalent), invasiveness, and long-term cost burden.

In this study, the primary aim was to investigate how substrate selection influences the resonant response and sensitivity of electromagnetic biosensors for non-invasive glucose monitoring. Table 9 summarizes the most commonly used rigid and flexible substrates, highlighting how trade-offs between dielectric loss, relative permittivity, and mechanical flexibility govern key performance metrics in RF sensor development.

In the present work, FR-4 and high-permittivity Rogers laminates were selected because they provide high electromagnetic performance for non-invasive glycaemia tracking; however, their mechanical rigidity limits direct use in wearable, long-term skin-mounted devices. As future work, the proposed architecture will be translated to flexible substrates, such as Rogers R/flex3600 and Kapton^®^ polyimide film, to enable conformal deployment while preserving favorable EM performance for biomedical applications.

## 6. Conclusions

This study demonstrates that substrate selection is a primary determinant of the dominant sensing observable and overall performance in planar microwave glucose biosensors. Using a hexagonal sixteen-cell DHC-CSRR array implemented on FR-4 and Rogers RO3210, results showed that both sensors behave as two-port band-stop filters near 3 GHz, but with distinct sensing modes: the low-loss, high-permittivity Rogers laminate enhances amplitude-based sensitivity, whereas FR-4 favors frequency-shift sensitivity, as confirmed by in vitro experiments and in vivo tests. Building on these insights, we implemented a compact AD8302-based readout that replaces the bulky VNA and demonstrated high agreement with capillary reference measurements in 31 participants, with all points falling in Clarke Error Grid Zone A and errors within a few mg/dL over the normoglycemic range. Across three repeated measurements per volunteer on the Rogers architecture, the overall amplitude uncertainty was approximately ±0.5 dB, and analysis of body weight, height, and age revealed negligible correlation with the inferred glucose values (R^2^ ≈ 0.002–0.004), indicating that these demographic factors did not act as secondary confounders. Future work will extend validation to larger cohorts that include prediabetic and diabetic subjects over a broader glycemic range, and translate the architecture to flexible, biocompatible substrates such as Rogers R/flex 3600 to enable wearable implementations. Potential long-term calibration drift and sensor degradation under repeated use were not assessed in the present study and will be addressed in dedicated longitudinal investigations. Finally, integrating the compact prototype’s calibration data with machine-learning regression models will be explored to further reduce prediction error and support real-time, portable non-invasive glucose monitoring in practical biomedical applications.

## Figures and Tables

**Table 1:** Coefficients of the single-pole Debye dispersion model [30].

Debye Parameter	Model Equation
$\epsilon_{\infty }\left(\chi \right)$	5.38 + 30 × 10^−3^·χ
$\epsilon_{stat}\left(\chi \right)$	80.68 − 0.207 × 10^−3^·χ
$\tau \left(\chi \right)$	9.68 + 0.23 × 10^−3^·χ (ps)

**Table 2:** Permittivity and loss tangent for different glucose concentrations (χ).

Glucose Samples	C1	C2	C3	C4	C5	C6
$\varepsilon_{r}^{\prime }$	78.275	78.295	78.315	78.395	78.602	78.949
tan(δ)	0.133	0.131	0.129	0.124	0.108	0.082

**Table 3:** Dimensions of the Two-Cell Hexagonal CSRR Structure on Rogers and FR-4 Substrates.

Substrate	D_Outer_	D_Inner_	Thickness (T)	Height (H)
Rogers	6.08 mm	4.61 mm	0.37 mm	1.27 mm
FR-4	7.60 mm	5.75 mm	0.46 mm	0.88 mm

**Table 4:** Comparison of sensitivity and R^2^ for FR-4 and Rogers substrates in one- and two-finger glucose sensing.

Configuration	Substrate	Δ∣S_21_∣ (dB)	Sensitivity dB/(mg/dL)	R^2^
One Finger	FR-4 Rogers	0.29	7.54 × 10^−3^	0.947
		1.16	1.22 × 10^−2^	0.993
Two Fingers	FR-4 Rogers	1.62	2.78 × 10^−2^	0.985
		6.82	9.35 × 10^−2^	0.973

**Table 5:** Statistical summary of the resonant ∣S_21_∣ amplitude measurements at 1.58 GHz.

Glucose Concentration (mg/dL)	Mean S_21_ (dB)	Standard Deviation (dB)	$U_{95\%}$ (dB)
60	−5.898	0.284	0.706
70	−6.638	0.239	0.595
90	−9.456	0.181	0.448
150	−12.777	0.162	0.402

**Table 6:** Comparison of state-of-the-art microwave glucose sensors based on S-parameter variation.

Sensing Technique	Frequency (GHz)	Test Method	GC (mg/dL)	Sensing Parameter	Sensitivity dB/(mg/mL)
Six CSRR Cells [26]	2.5–3.5	Aqueous Solution	0–160	S_21_	7.47 × 10^−3^
Triangular CSRR [37]	2.1	Aqueous Solution	50–200	S_21_	7.10 × 10^−2^
Triple Pole CSRR [44]	2.3	Synthesis Blood Samples	70–120	S_21_	6.20 × 10^−2^
Dielectric Waveguide Probe [45]	2.0–2.5	Aqueous Solution	0–30,000	S_11_	3.00 × 10^−5^
Microstrip line [46]	1.4–1.9	Aqueous Solution	78–5000	S_11_	1.80 × 10^−3^
T-patch CSRR [47]	1.02–2.24	Aqueous Solution	0–594	S_11_	1.60 × 10^−3^
Single Port Sensor [48]	2.0–3.0	Aqueous Solution	0–20,000	S_11_	6.40 × 10^−2^
Three-Ring SRR [49]	2.4–5.2	Single-Finger	0–400	S_11_	1.10 × 10^−2^
This Work	1.5	Dual-Finger	60–150	S_21_	9.36 × 10^−2^

**Table 7:** Baseline Characteristics of the cohort (*n* = 31). Data are presented as mean ± SD or *n* (%).

Characteristic	Value
Age (years)	22.3 ± 3.3
Height (cm)	173.9 ± 9.8
Weight (kg)	76.6 ± 11.5
BMI (kg/m^2^)	25.3 ± 2.9
Glucose (mg/dL)	90.8 ± 16.5
Male, *n* (%)	19 (61.3%)
Female, *n* (%)	12 (38.7%)

**Table 8:** Benchmarking the proposed non-invasive microwave sensor against commercial glucose monitoring systems.

System	Accuracy (MARD or Equivalent)	Sensitivity/Dynamics	Invasiveness	Approximate Cost/Burden
Dexcom G7 (iCGM) [50]	MARD ≈ 8.2% [51]	5 min continuous readings; hypo/hyper alarms; non-adjunctive dosing	Minimally invasive 10-day subcutaneous filament	High recurring sensor cost; typically, several hundred USD/month
FreeStyle Libre 3 [52]	>90% of readings within ±20%/20 mg/dL [53]	1 min readings to smartphone; optional alarms	Minimally invasive, worn up to 14 days [54]	Lower-cost CGM; ≈ USD 70–80 per sensor
Medtronic Guardian 4 [55]	MARD ≈ 8.2–8.5% [56]	5 min readings; predictive low-glucose management in hybrid closed-loop pumps	Minimally invasive 7-day subcutaneous sensor + transmitter [56]	Recurring sensor costs comparable to other CGMs
Eversense E3 [57]	MARD ≈ 8.5–8.8% over 180 days [58,59]	Continuous long-term CGM; on-body vibrating alerts and app trends	Implantable optical sensor in the upper arm (6 months) + external transmitter [60]	Higher annual cost, including insertion/removal procedures; fewer insertions per year
Capillary finger-stick glucometer [61]	98.8% of readings within ±15 mg/dL or ±15% [62]	High single-point accuracy; no continuous trend; depends on test frequency [63]	Fully invasive repeated finger-prick sampling	Low device cost: ongoing strip cost dominates, highly payer-dependent
Proposed dual-finger DHC-CSRR sensor (this work)	97.83%; 100% within ±15 mg/dL (ISO 15197) and in Clarke zone A (60–125 mg/dL)	Non-invasive amplitude-based readout; local sensitivity ≈1.099 mg/dL/mV and ≈0.0935 dB/(mg/dL)	Fully non-invasive dual-finger contact on a planar microwave structure; no skin penetration	Research prototype; planar RF hardware with no per-sensor consumables, only periodic calibration

**Table 9:** Representative rigid and flexible substrates commonly used in non-invasive electromagnetic glucose sensing [64].

Type	Substrate Material	$\mathrm{Typical}\;\varepsilon_{r}$ (1–10) GHz	Typical Thickness Range (mm)	Main Advantages	Main Limitations
Rigid	FR-4 epoxy–glass laminate	3.8–4.5	0.8–2.0 (1.6 mm standard)	Very low cost; compatible with standard PCB fabrication; suitable for proof-of-concept resonators and antennas	high dielectric loss, reducing Q-factor and sensitivity; ε and loss vary with batch and moisture; limited suitability for precision metrology.
Rigid	Rogers RO4350B (hydrocarbon/ceramic)	3.3–3.7	0.10–1.52	Low-loss supports high-Q resonators and stable S-parameter measurements.	Higher cost than FR-4; rigid and non-conformal, limiting direct on-skin or curved-body placement
Rigid	Rogers RO3210 (ceramic-filled PTFE)	10.2	0.25–1.27	Very high ε_r_ enabling compact resonators and strong near-field confinement; enhances sensitivity to small permittivity changes.	Narrower impedance bandwidth, more sensitive to fabrication tolerances; rigid and higher material cost.
Flexible	Rogers R/flex 3600 (LCP foil)	2.6–2.7	0.025–0.050	Flexible, low-loss, and moisture-stable, suitable for conformal on skin for CGM.	Requires specialized flexible-circuit processing; higher cost and limited availability than standard rigid laminates.
Flexible	Kapton^®^ polyimide film	3.2–3.5	0.025–0.125	Thermally stable low-loss flexible film; widely used in printed circuits and lightweight wearable sensor prototypes.	Bendable but not stretchable; repeated bending can induce micro-cracks and resonance detuning over time.
Flexible	PDMS (polydimethylsiloxane)	2.6–3.0	0.5–2.0 (cast layers)	Soft, skin-conformal and biocompatible; excellent for on-skin coupling and integration of microfluidic channels with glucose solutions or sweat.	Higher dielectric loss than LCP or polyimide; thickness uniformity depends on the casting process; long-term creep can gradually shift resonance.
Flexible	Textile substrate (e.g., denim)	1.6–1.8	1.0–2.5	Intrinsically wearable and breathable, enables garment-level integration of antennas.	ε_r_ and loss are strongly affected by sweat, humidity, and pressure; poor repeatability

## Data Availability

The original contributions presented in this study are included in the article. Further inquiries can be directed to the corresponding authors.

## Nomenclature

Latin symbols	
A,B,C	Coefficients of the quadratic voltage–to–glucose calibration $G(V)=AV^{2}+BV+C$
b	Intercept of the regression between predicted and reference glucose (mg/dL)
CI	Confidence interval of the mean
$D_{\mathrm{out}}$	Diagonal length of outer hexagonal CSRR cell (mm)
$D_{\mathrm{in}}$	Diagonal length of inner hexagonal CSRR cell (mm)
$e_{i}$	Residual error for sample i, $e_{i}=y_{\mathrm{pred},i}-y_{\mathrm{refr},i}$(mg/dL)
$f_{0}$	Fundamental resonant frequency (Hz)
$f_{1}$	Second-mode resonant frequency (Hz)
G	Blood glucose concentration (mg/dL)
$\hat{G}$	Predicted glucose concentration from regression model (mg/dL)
H	Substrate height/thickness (mm)
k	Number of groups (participants) in pooled–variance analysis
m	Slope of the regression between predicted and reference glucose (dimensionless)
n	Number of repeated measurements per participant
N	Total number of samples in the dataset
Q	Resonator quality factor, $Q=f_{0}/\Delta f_{3\mathrm{dB}}$(dimensionless)
$R_{\varepsilon }$	Amplitude resolution with respect to change in real permittivity, $\Delta |S_{21}|/\Delta \varepsilon_{r}$(dB)
$R_{\tan\delta }$	Amplitude resolution with respect to change in loss tangent, $\Delta |S_{21}|/\Delta tan\delta$(dB)
s	Sample standard deviation of repeated $|S_{21}|$measurements (dB)
$s_{p}$	Pooled standard deviation across participants (dB)
|S_21_|	Magnitude of the transmission coefficient (dB)
Δ|S_21_|	Change in |S_21_| with glucose or dielectric properties (dB)
$\Delta f_{3\mathrm{dB}}$	3 dB bandwidth around the resonance (Hz)
SEM	Standard error of the mean, $s/\sqrt{n}$(dB)
$U_{95\%}$	Expanded uncertainty of the mean at 95% confidence (dB)
V	Output voltage of the AD8302 board/sensor chain (V)
$\bar{V}$	Mean voltage across all participants (V or mV)
$\bar{x}$	Mean |S_21_| amplitude for repeated measurements at a given glucose level (dB)
$y_{\mathrm{pred},i}$	Predicted glucose for the sample i(mg/dL)
$y_{\mathrm{refr},i}$	Reference the glucometer glucose for the sample i(mg/dL)
Greek symbols	
$\varepsilon_{r}$	Complex relative permittivity, $\varepsilon_{r}=\varepsilon_{r}^{\prime }-j\varepsilon_{r}^{\prime \prime }$(dimensionless)
$\varepsilon_{r}^{\prime }$	Real part of relative permittivity (energy storage) (dimensionless)
$\varepsilon_{r}^{\prime \prime }$	Imaginary part of relative permittivity (loss) (dimensionless)
$\varepsilon_{\mathrm{stat}}$	Static (low-frequency) permittivity in Debye model (dimensionless)
$\varepsilon_{\infty }$	High-frequency permittivity limit in Debye model (dimensionless)
χ	Glucose concentration variable in Debye model (mg/dL)
δ	Dielectric loss angle
tanδ	Loss tangent, $\varepsilon_{r}^{\prime \prime }/\varepsilon_{r}^{\prime }$(dimensionless)
ω	Angular frequency, ω=2πf(rad/s)
τ	Debye relaxation time (ps)
ν	Degrees of freedom for Student’s t–distribution (ν=n−1)
Statistical and error metrics	
MAE	Mean absolute error of glucose prediction (mg/dL)
MAPE	Mean absolute percentage error of glucose prediction (%)
MARD	Mean absolute relative difference between predicted and reference glucose (%)
RMSE	Root–mean–square error of glucose prediction (mg/dL)
SDAE	Standard deviation of absolute errors (mg/dL)
Abbreviations and acronyms	
AD8302	Analog Devices gain/phase detector IC
BGL	Blood glucose level
BMI	Body mass index
CGM	Continuous glucose monitoring
CSRR	Complementary split ring resonator
DHC–CSRR	Double-honeycomb complementary split ring resonator (16–cell array)
DMM	Digital multimeter
GC	Glucose concentration
ISF	Interstitial fluid
MI	Minimally invasive
NI	Non-invasive
QF	Quality factor
RF	Radio frequency
RO3210	Rogers RO3210 substrate
VNA	Vector network analyzer
